# *In Vitro* Filaricidal Properties of Aqueous Extracts of *Combretum nigricans* (Combretaceae) on *Onchocerca ochengi* (Onchocercidae)

**DOI:** 10.1155/2024/2119056

**Published:** 2024-01-31

**Authors:** Banserne Brey Ignagali, Borris Rosnay Galani Tietcheu, Theodore Betrosse, Blaise Kamaya, Dieudonne Ndjonka

**Affiliations:** Laboratory of Applied Biochemistry, Department of Biological Sciences, Faculty of Science, University of Ngaoundere, Ngaoundere, Cameroon

## Abstract

**Aim:**

Onchocerciasis is an endemic parasitic disease in sub-Saharan Africa that significantly impacts animal and human health. In Northern Cameroon, medicinal plants from the *Combretum* genus are used for onchocerciasis traditional treatment although there is no scientific evidence of their antifilarial potential. This study evaluates the *in vitro* macro- and microfilaricidal properties of water extracts from *Combretum nigricans* in *Onchocerca ochengi*. *Material and Methods*. *O. ochengi* microfilariae and adult male worms were recovered from cowhide fragments. Oxidative stress indicators and motility tests were used to assess the filaricidal impact. Female albino rats were used to test for acute toxicity. The contents of secondary metabolites were quantified.

**Results:**

The bark aqueous extract was more active on macrofilariae at 1 mg/mL for 24 h (100%) than the leaf (63.9%) and root (75%) extracts at the same concentration. Likewise, a stronger microfilaricidal effect was found with this extract at 0.5 mg/mL for 1 h (100%) compared to root and leaf extracts. The dose-response effect with the bark extract gave an inhibitory concentration 50 (IC_50_) of 351 *μ*g/mL vs. 113 *μ*g/mL for flubendazole after 24 h incubation, while the microfilaricidal efficacy revealed an IC_50_ of 158.7 *μ*g/mL vs. 54.09 *μ*g/mL for ivermectin after one-hour incubation. Examining stress indicators on parasite homogenates showed that macrofilaricidal activity is associated with a significant increase in nitric oxide, glutathione, and malondialdehyde generation and a decrease in catalase activity. At 2000 mg/kg, rats showed no harm. The phytochemical investigation revealed that the barks contained more phenolic acids, condensed tannins, flavonoids, and saponins than the leaves (*p* < 0.001).

**Conclusion:**

These findings support *C. nigricans*' antifilarial activity and identify oxidative stress indicators as prospective treatment targets in *O. ochengi*. It would be interesting to conduct in vivo studies to understand their antifilarial activity better.

## 1. Introduction

Helminthiasis is a severe public health concern that affects both humans and animals worldwide [1]. The three primary helminth groups that cause this disease include nematodes, cestodes, and trematodes. According to recent World Health Organization (WHO) estimates, around 1.5 billion helminth infections occur globally [2]. Nematodes of the *Filarioidea* superfamily specifically induce helminthiases known as filariasis. In 2018, lymphatic filariasis was found in 51 million people worldwide [3] while 220 million people were found exposed to onchocerciasis in 2017 [4].

With 20.9 million cases globally, *Onchocerca volvulus* filariasis is quite common; of those, 14.6 million have skin conditions and 1.15 million have vision impairments [4]. Approximately 99% of those infected globally reside in 30 sub-Saharan African nations. Of the 37 million affected, 270,000 have skin problems and 500,000 have vision problems [5]. In Cameroon, several areas have differing rates of endemicity for onchocerciasis. Over 60% of individuals live in high-risk areas, 2.8 million people are infected, and 5.2 million people are in danger of infection [6]. A prevalence of 10.7% and 6.7% in men and women, respectively, was recorded after seven years of continuous community-directed treatment of skin conditions with ivermectin in the central region [7]. A study carried out in southern Cameroon showed that out of 9,456 subjects diagnosed, the prevalence of onchocerciasis was 44.4% [8]. In the Adamaoua region, the prevalence of human and bovine onchocerciasis is estimated at 30% and 65%, respectively [9].

When a healthy man gets bitten by an infected black fly, this latter injects infectious larvae known as L3-stage larvae, which eventually become adults and release microfilariae after penetrating the subcutaneous tissues. Subsequently, they may extend to the eye and deeper organs, hence causing cutaneous and ocular manifestations including long-term irreversible blindness [10].

Conventionally, onchocerciasis is treated with ivermectin and moxidectin. However, these drugs are mainly microfilaricidal and have negative side effects that include leukopenia, nausea, vomiting, and diarrhea. In addition to the parasites' development of resistance, a possible tissue reinfestation by microfilariae is not to be ruled out [11]. Anti-Wolbachia therapy has recently emerged as a promising approach against onchocerciasis, thanks to the ability of antibiotics such as doxycycline or azithromycin to exert potent macro-filaricidal activities and impede embryogenesis in adult female worms [12]. However, these medications are not permitted in pregnant women or children under the age of nine. Other promising alternatives, such as rapamycin, berberine, and globomycin [12], or high-dose rifampicin and ABBV-4083 [13], are in the clinical phases. New studies recently showed that the triple combination (ivermectin/diethylcarbamazine/albendazole) outperformed the double combination (ivermectin/albendazole) in eliminating or inactivating female *O. volvulus* worms and is well-tolerated [14]. However, the macrofilaricidal effect observed was partial, requesting thereby additional combination studies. Finding antifilarial drugs that are both potent macro- and microfilaricidal agents is crucial and urgent given the range of treatment-related issues. Medicinal plants, therefore, appear like an alternative solution as a number of them demonstrated intriguing antionchocercal qualities [15].

*Combretum nigricans* is a plant of the *Combretaceae* family traditionally used in sub-Saharan Africa as a remedy against gastrointestinal ailments [16] and to treat conjunctivitis, cataracts, icterus, and rheumatism [17]. Previous studies reported anticancer properties with triterpenes extracted from the leaf methanolic extract [18] and anthelminthic activities with the organic and aqueous extracts of fruits against the levamisole-resistant strain of *Caenorhabditis elegans* [19]. Other investigations found antimalarial [20], antianemia [21], and antifungal [22] activities. However, little information is available on their filaricidal activities, yet a recent ethnobotanical study showed that the populations of Far North Cameroon use *Combretum* species to treat onchocerciasis [23]. The general objective of this present study consisted of experimentally evaluating the *in vitro* antifilarial properties of some aqueous extracts of *Combretum nigricans* on *Onchocerca ochengi*.

## 2. Materials and Methods

### 2.1. Plant Collection and Identification

The leaves, stem bark, and roots of *Combretum nigricans* were harvested in April 2023 in Ngaoundéré in the Adamaoua region of Cameroon as previously described by earlier reports [20, 24]. Plant identification was carried out by Dr. Fawa Guidawa, a botanist from the Faculty of Sciences of the University of Ngaoundéré. The different parts were washed in tap water and dried away from sunlight and humidity for three weeks. Then, the plant material was crushed in a traditional wooden mortar. Finally, the powders were kept in the boxes until the extracts were prepared.

### 2.2. Preparation of Aqueous Extracts of *Combretum nigricans*

Twenty grams (20 g) of powder from each plant part was introduced into 200 mL of distilled water, and the mixtures were brought to boil for twenty (20) minutes using a hotplate. After cooling, the decoctions were filtered using Whatman 4 filter paper, and the filtrates obtained were placed in an oven for evaporation at 40°C for two days. The crude aqueous extracts were weighed using a sensitive balance and then stored at room temperature for further study. Percentage yields were estimated as previously described [25] by making the ratio of the mass of the dry crude extract^∗^100/mass of the powder.

### 2.3. Onchocerca Ochengi Worms' Isolation

#### 2.3.1. Adult Male Worms

Twenty pieces of nodule-infested fresh skins were collected from 10 cows (either 2 skin/cow) at the municipal slaughterhouse of Ngaoundere II. The entire skin was removed using a knife after careful palpation to check for the presence of nodules at the cow's udder, a site of predilection for *O. ochengi*. The infected skin pieces were brought to the laboratory and washed with tap water, bleach, and soap. The different internal and external faces of each piece of skin were sterilized with 70% ethanol by spraying. Individual *Onchocerca* nodules were isolated from the skin using a scalpel fitted with a scalpel blade by making slight incisions on the inner side of the skin. Isolated nodules were preserved in the sterile-filtered Roswell Park Memorial Institute 1640 (RPMI-1640) culture medium containing 25 mM 4-(2-hydroxyethyl)-1-piperazine ethane sulfonic acid and L-glutamine (GeneDirex, Inc., Taoyuan County, Taiwan) in the Petri dish and subsequently dissected. To dissect, a nodule is grasped by the edges of thick forceps, a small incision is made on the nodule wall, and light pressure on the nodule allows the nodule contents to exit into the RPMI-1640. These were incubated in the incubator at 37°C for 30 minutes. Under a binocular magnifying glass, the highly mobile male worms were distinguished from the females and isolated using fine forceps and a mounted needle and then placed in the RPMI-1640 [26].

#### 2.3.2. Microfilariae

The washed skin pieces were first shaved with a razor blade and rinsed in distilled water. Excess moisture was removed with a sterile pad and then covered with 70% ethanol. Pieces of superficial skin tissues were cut and put into the culture medium and incubated for 4 to 6 hours. The very mobile emerged microfilariae were concentrated by centrifugation at 400 rpm for 20 minutes, and finally, the number of microfilariae was counted under a binocular magnifying glass as reported.

### 2.4. Survival of Adult Male Worms and Microfilariae under Laboratory Conditions

After isolation, the adult worms were examined under a binocular microscope to differentiate between injured and healthy worms. Injured worms were simply removed to ensure that all cultured worms were healthy and viable. To determine the optimal motility duration of these healthy worms, they were washed twice in RPMI-1640 medium (6 worms cultured in 1 mL of RPMI-1640 medium) and incubated at 37°C in a humidified atmosphere for 96 h. Two tests with 3 repetitions were carried out in 24-well plates; then, the average of the motility inhibition rates was calculated. Similarly, microfilariae were incubated at a density of 17 microfilariae/well under such conditions, and motility tests were performed every 2 h [27] for 10 h. Worms were monitored for 15 seconds apiece throughout the motility testing. Worms that were immobile for more than 15 seconds were considered dead, and those that moved were regarded as alive.

### 2.5. Antifilarial Activities of Aqueous Extracts of *Combretum nigricans*

#### 2.5.1. Antifilarial Screening on Mfs and mfs

Firstly, screening was carried out on macrofilariae (Mfs) at a single concentration of 1 mg/mL of each plant part extract to focus on the most active extract. The crude extract with the best *in vitro* efficacy was selected for dose-response studies. Adult male *O. ochengi* worms (6 worms/well) were incubated at 37°C in 24-well plates and for 24 h with 1 mg/mL of plant extract in the RPMI-1640 medium. Flubendazole (0.3 mg/mL)-treated worms were used as the positive control while nontreated worms were used as the normal control. Regarding the microfilaricidal tests, microfilariae (17 mfs/well) were incubated in 24-well plates for 4 h in the absence (normal control) or the presence of 0.5 mg/mL of each plant part extract (tests groups) or ivermectin at 0.2 mg/mL (positive control). Ivermectin was preferred over flubendazole as a positive control for mfs because previous research indicated it to be more active on juvenile worms than adult worms [28]. After the incubation time, motility was assessed under a binocular microscope as earlier shown [29].

#### 2.5.2. Dose-Response Study

The aqueous extract which showed the most interesting activity (100%) during the screening on adult male worms and microfilariae was tested at increasing concentrations (0-700 *μ*g/mL on Mfs with a step of 100 *μ*g/mL and 0-300 *μ*g/mL on mfs with a step of 50 *μ*g/mL) to determine the values of the half-inhibitory concentrations (IC_50_). Positive controls were carried out with flubendazole (Mfs) and ivermectin (mfs) in the same conditions. The normal control was carried out only with RPMI-1640 culture medium.

### 2.6. Evaluation of Oxidative Stress Parameters in the Homogenates of Adult Male Worms

After 24 h of incubation, the supernatant was removed from the culture plate and the adult male *O. ochengi worms* were washed twice in the phosphate buffer saline (PBS). Then, 1 mL of fresh PBS was added, and grinding was carried out on a Petri dish using a scalpel. The mixture was removed and centrifuged at 3500 rpm for 10 minutes, and finally, the supernatant was collected, stored in the refrigerator, and subsequently used for the different assays [27]. Quantification of levels of glutathione (GSH) [30], nitric oxide (NO) [31], malondialdehyde (MDA) [32], and catalase (CAT) activity [33] was carried out by spectrophotometric assay.

#### 2.6.1. GSH Content Determination

The Ellman reagent (1500 *μ*L) was introduced into test tubes previously containing 100 *μ*L of plant extract (test tubes) and 100 *μ*L of tris-HCl phosphate buffer (50 mM, pH 7, 4/Mc-Even buffer) (control tube). The tubes were shaken for 30 minutes at room temperature, and then, the absorbance of the samples was read on the spectrophotometer at 412 nm against the blank.

#### 2.6.2. NO Assay

The Griess reagent (Biotium, Fremont, USA) (500 *μ*L) plus 100 *μ*L of the sample was combined in a cuvette. 400 *μ*L of distilled water was added, and then, the mixture was incubated for 10 minutes at room temperature. Preparation of a reference sample was done by adding 500 *μ*L of the Griess reagent into 500 *μ*L of deionized water. The absorbance measurement of the sample containing nitrite was observed at 546 nm relative to the reference sample using a spectrophotometer. A standard was prepared with a sodium nitrite solution with increasing concentrations by diluting the nitrite standard solution with distilled water [31].

#### 2.6.3. CAT Assay

Two tubes A (blank) and B (sample) were used. In tube A, 50 *μ*L of distilled water, 750 *μ*L of 0.1 mM phosphate buffer, pH 7.5, and 200 *μ*L of hydrogen peroxide were introduced. In the second tube, 50 *μ*L of the homogenates, 750 *μ*L of 0.1 mM phosphate buffer, pH 7.5, and 200 *μ*L of 50 mM hydrogen peroxide were introduced. Then, both tubes were incubated for 1 minute at room temperature, and 2000 *μ*L of potassium dichromate/glacial acid was added to each tube. Both tubes were capped using glass beads, and the solutions were heated to 100°C for 10 minutes. After cooling, the absorbance was read against the blank using a spectrophotometer at 570 nm.

#### 2.6.4. MDA Assay

Two tubes A (reagent blank) and B (sample) were used. In tube A, we introduced 250 *μ*L of tris-HCl phosphate buffer (50 mM, pH 7.4/Mc-Even buffer), 125 *μ*L of 20% trichloroacetic acid (TCA), and 250 *μ*L of 0.67% thiobarbituric acid (TBA). In the second tube, 250 *μ*L of the homogenates, 125 *μ*L of 20% TCA, and 250 *μ*L of 0.67% TBA were introduced. Both tubes were capped using glass beads, and the solutions were heated to 90°C in a water bath for 10 minutes. After cooling in tap water, the tubes were centrifuged at 3000 rpm at room temperature for 15 minutes. The supernatant was pipetted, and the absorbance was read against the blank using a spectrophotometer at 530 nm.

### 2.7. Acute Toxicity to Female Albino Rats of the Wistar Strain

Twelve female Wistar albino rats weighing 150-180 g were obtained from the animal house at the National School of Agro-Industrial Sciences of the University of Ngaoundere, Adamaoua, Cameroon. The animals were kept at a room temperature of 25 ± 2°C and on a 12/12 h light/dark cycle, and they were fed a conventional rat diet and allowed free access to tap water.

The oral acute toxicity of *C. nigricans* extracts (leaves, bark, and roots) was carried out following the 423 Organization for Economic Co-operation and Development guidelines for testing chemicals [34]. The female albino rats were deprived of food but not water for 15 h, weighed after fasting, and divided into 4 groups of 3 rats each (3 test groups and one control group). Before administration, the extract was dissolved in distilled water to a final concentration of 200 mg/mL corresponding to a body weight dose of 2000 mg/kg. Then, the extracts were administered orally to the animals in the tested group. The bark extract was given to the first test group at a dose of 50 mg/kg, the second group at a dose of 300 mg/kg, and the third group at a dose of 2000 mg/kg. All groups were deprived of food for 3 hours after treatment. On the other hand, those in the control group only received a standard administration volume of 10 mL/kg of distilled water. We observed signs such as piloerection, the appearance of stools, drowsiness, motility, grooming, sensitivity to noise, and mortality in animals every 4 h on the first day and then twice daily on the following days for 14 days. Weight changes were recorded weekly during the observation period.

### 2.8. Ethical Approval

This study was conducted following the ASAB Ethical and ABS Animal Care Committee recommendations for the ethical treatment of nonhuman animals in behavioral research and education [35]. All animal experiments were reviewed and approved by the Department of Biological Sciences of the Faculty of Science of the University of Ngaoundere (Cameroon).

### 2.9. Phytochemical Study

#### 2.9.1. Qualitative Phytochemical Screening

*Combretum nigricans* aqueous extracts (leaves, roots, and bark) were subjected to phytochemical analysis to highlight potential antifilarial secondary metabolites. Qualitative tests were carried out using standard staining methods to identify phenolic compounds, flavonoids, alkaloids, anthocyanins, tannins, triterpenes, glucosides, and saponins. Briefly, to demonstrate the presence of alkaloids, 1 mL of 5% HCl and 3 drops of the Dragendorff reagent were added to 1 mL of extract, and the orange color was considered a positive test [36]. Regarding flavonoids, 1 mL of each extract was added to 100 *μ*L of concentrated HCl and a few magnesium shavings. The presence of flavonoids was confirmed by the appearance of red or orange color [37]. The presence of tannins indicated by a greenish or blue-black coloring was detected using the ferric chloride test [37]. Phenolic acids were detected by adding three drops of 10% ferric chloride to the extract; the formation of a greenish color was considered as a positive reaction. Saponins were highlighted by shaking 10 mL of the extract in water for 15 seconds and letting stand for 15 minutes. A persistent moss height, greater than 1 cm, indicated a positive reaction [36]. Triterpenes were identified using the Salkowski test as reported earlier [38]. In brief, 5 mL of the extract was added to 2 mL of chloroform and 3 mL of concentrated sulfuric acid. The formation of a brown-red ring at the interface was regarded as a positive reaction. Glucosides, deoxyoses, and anthocyanins were detected as described by Himour et al. [39].

#### 2.9.2. Quantitative Phytochemical Screening

Phenolic acids were quantified as previously reported [40]. Briefly, 100 *μ*L of extract (100 *μ*g/mL) to be analyzed was mixed with 500 *μ*L of the Folin-Ciocalteu reagent (10%). Then, 400 *μ*L of a sodium carbonate solution (7% *w*/*v* Na_2_CO_3_) has been added. After shaking, the whole was incubated in the dark, at room temperature for 10 minutes. The absorbance was read at 760 nm using a UV spectrophotometer against a blank. The content of phenolic compounds was expressed as milligrams of gallic acid equivalents per gram of dry plant material (mg GAE/g DM).

Regarding condensed tannins, 200 *μ*L of the extract (100 *μ*g/mL) was added to 1000 *μ*L of the vanillin reagent previously maintained at 30°C; then, the whole was shaken and incubated for 20 min for darkness at room temperature. The absorbance was measured at 500 nm by a UV spectrophotometer against a blank consisting of a mixture of equal volumes of methanol (37%) and 8% HCl. The condensed tannin content of the extracts was expressed in milligrams of catechin equivalents per gram of extract (mg CE/g E) [41].

As far as flavonoids are concerned, 1 mL of sample (100 *μ*g/mL) or rutin was added to 1 mL of AlCl_3_ (2%, m/v in MeOH). After brief shaking, followed by incubation for 30 minutes at room temperature and protection from light, the absorbances were read with a spectrophotometer at 430 nm. The total flavonoid content was determined from a regression line and expressed in milligrams of rutin equivalents per gram of dry plant matter (mg RE/g DM) [42].

### 2.10. Statistical Analyses

The data obtained were presented as mean values ± standard error of the mean (SEM). Graphical analyses and IC_50_ values were determined using GraphPad Software (version 8.0.1). The Shapiro-Wilk test was used to check normality. Comparisons between antioxidant parameters, macro- and microfilaricidal activities, and secondary metabolite contents were carried out using a one-way analysis of variance followed by Dunnett's multiple comparison test. Statistical difference was declared at *p* < 0.05.

## 3. Results

### 3.1. Extraction Yields

The extraction yields of leaves, barks, and roots are displayed in Table 1. As shown by this table, the yields of the aqueous extracts of the roots (5%) and leaves (6.5%) of *C. nigricans* are lesser than those of the trunk barks (10%).

### 3.2. Survival Rate of Macrofilariae and Microfilariae in the Incubation Conditions

**Figure 1:**
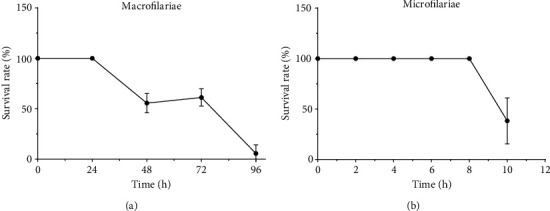
Survival rates of (a) adult male worms and (b) microfilariae in RPMI-1640 culture medium. Adult worms and microfilariae were incubated in the RPMI-1640 medium at 37°C in a humidified atmosphere for 96 h and 10 h, respectively. Then, the inhibition motility tests were carried out under a binocular microscope, and the survival rates were determined. Data were expressed in means ± SEM.

Figure 1(a) shows that adult male worms were all motile for 24 h (100%) under the incubation conditions. The motility started decreasing from 48 h (55.6 ± 5.56%) until reaching the lowest value at 96 h (5.54 ± 3.54%) of incubation. The mfs were however all mobile for 8 h (100%). Their motilities dropped from 10 h (38.23 ± 11.39%) (Figure 1(b)).

### 3.3. Macro- and Microfilaricidal Activities of Aqueous Extracts of *Combretum nigricans* on *Onchocerca ochengi*

**Figure 2:**
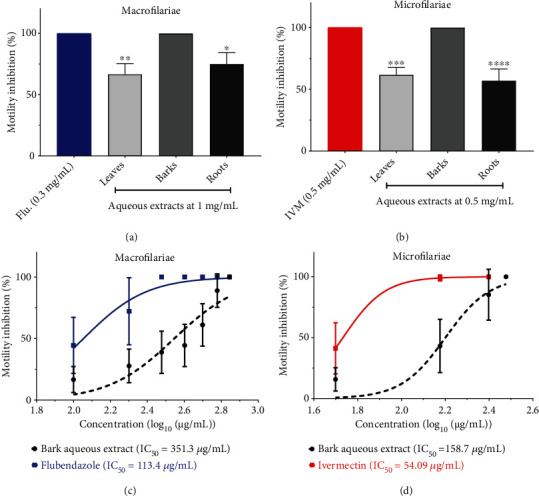
Macro- and microfilaricidal activities of various aqueous extracts of *Combretum nigricans* on *Onchocerca ochengi* in comparison to that of flubendazole and ivermectin and dose-response curves. Adult worms (6/well) were incubated in 24-well plates in the RPMI-1640 medium at 37°C in a humidified atmosphere in the presence of flubendazole (Flu.) at 0.3 mg/mL or the aqueous extracts of leaves, barks, and roots at 1 mg/mL for 24 h, and the dose-response study was conducted to record the inhibition concentration 50 (IC_50_) values. Microfilariae (17/well) were incubated with the plant extracts or ivermectin (IVM) at 0.5 mg/mL for the screening, and the IC_50_ was determined using motility inhibition tests. Data were expressed in means ± SEM. ^∗^*p* < 0.05, ^∗∗^*p* < 0.01, ^∗∗∗^*p* < 0.001, and ^∗∗∗∗^*p* < 0.0001 when compared with the flubendazole or the ivermectin-treated group.

The macrofilaricidal screening of the different plant aqueous extracts at 1 mg/mL showed, after 24 h incubation a stronger inhibition of motility with the bark extract (100 ± 0.00%) compared to other extracts. Indeed, the leaf (63.9 ± 7.95%, *p* < 0.01) and the root extracts (75 ± 9.38%, *p* < 0.05) (Figure 2(a)) displayed a significantly lower macrofilaricidal activity than flubendazole. However, the effect of the bark extract was comparable to that of flubendazole 0.3 mg/mL (*p* > 0.05). Similarly, the microfilaricidal screening revealed that the bark extract had a greater impact (100% at 0.5 mg/mL) during the first hour of incubation than other plant extracts, and this effect was identical to ivermectin at 0.5 mg/mL (Figure 2(b)).

A dose-response investigation was then performed with the bark extract on both adult and juvenile worms. Its IC_50_ values were found to be three times higher than those of flubendazole (351.3 *μ*g/mL vs. 113 *μ*g/mL) (Figure 2(c)) and ivermectin (158.7 *μ*g/mL vs. 54.09 *μ*g/mL) (Figure 2(d)), respectively, suggesting thereby a moderate efficacy.

### 3.4. Biochemical Effects Associated with the Macrofilaricidal Activity of the Aqueous Extract of the Bark of *Combretum nigricans* and Flubendazole

To get an insight into the metabolic changes induced by the bark extract in the treated worms, different markers of oxidative stress were investigated in worm homogenates. As shown by Table 2, the bark aqueous extract at 700 *μ*g/mL (3.31 ± 0.891 *μ*moL/mL) and 300 *μ*g/mL (1.72 ± 0.130 *μ*mol/mL) caused a nonsignificant and concentration-dependent increase in the GSH content as compared to the nontreated group (1.54 ± 0.005 *μ*mol/mL) similarly to flubendazole at 300 *μ*g/mL(2.60 ± 0.527  *μ*mol/mL. However, a significant increase (*p* < 0.001) in NO contents was observed with flubendazole at 300 *μ*g/mL (164 ± 21.8 mM/mL) and the bark extracts at 700 *μ*g/mL (172 ± 13.5 mM/mL) comparatively with the normal control (17.7 ± 13 mM/mL). The MDA content increased but not significantly (*p* > 0.05) at all concentrations of the bark extract and that of flubendazole. Flubendazole at 300 *μ*g/mL (32 ± 16.5 *μ*M/min, *p* < 0.05) and barks at 700 *μ*g/mL (88 ± 32 *μ*M/Min) decreased catalase activity (CAT) compared to the untreated group (100 ± 36 *μ*M/Min).

### 3.5. Analysis of Acute Toxicity on Female Albino Rats

Considering the moderate efficacy of the bark extract, we found it useful to explore the acute toxicity profile to get an insight into the selectivity. Administration of a single dose of 2000 mg/kg of aqueous extracts of *C. nigricans* to albino rats orally did not produce clinical signs of toxicity or mortality (Table 3).

### 3.6. Qualitative Phytochemical Profile

The presence of tannins, saponins, phenolic acids, flavonoids, anthocyanins, and deoxyoses was noted in the barks while alkaloids and phenolic acids were found in the roots and tannins, saponins, phenolic acids, triterpenes, glycosides, and deoxyoses were detected in the leaves (Table 4).

### 3.7. Quantitative Phytochemical Profile

Phenolic acids were quantitatively more important in the bark extracts (98.7 ± 0.402 mg GAE/g DM, *p* < 0.001) than in leaves (56.5 ± 0.26 mg GAE/g DM) and roots (8.79 ± 0.292 mg GAE/g DM) (Table 5). Condensed tannins were significantly lower in the leaves (475 ± 2.52 mg CE/g DM) extracts than in the bark (545 ± 0.333 mg CE/g DM) and roots (588 ± 4.63 mg CE/g DM). Regarding flavonoids, they were significantly more abundant in the bark (361 ± 1.35 mg RE/g DM) than in the roots (345 ± 1.49 mg RE/g DM) and leaves (226 ± 0.206 mg RE/g DM). Saponins (0.0612 ± 0.0139 mg) also were more abundant in the bark extract than in the other extracts.

## 4. Discussion

This study is aimed at evaluating the *in vitro* filaricidal properties of some water extracts of *C. nigricans* on an experimental model of onchocerciasis. First of all, the bark aqueous extract had the highest extraction yield (10%) compared to leaves and root extracts (Table 1), suggesting it concentrates more hydrosoluble secondary metabolites. This finding differs from previous studies that found a yield of 15.30% with aqueous extracts of *Combretum glutinosum* roots [43] and 5.70% with *Combretum micranthum* leaves [44]. Although all these plants belong to the same family, this difference could be explained either by the time and place of the harvest of each plant, the secondary metabolites present in each species, and even the climatic and environmental conditions or the volume of the solvent used and the mass of the vegetable powder.

Suitable conditions for the incubation of adult male worms and mfs were explored in the laboratory environment. Thus, the worm survival experiments were conducted in the RPMI-1640 culture medium in the absence of our various aqueous extracts. Adult male worms were found motile in the medium for 24 h while mfs were stable for 8 h of incubation at 37°C. These incubation periods are significantly shorter than those reported in a recent study by our research group, which found that the survival times of adult male worms and mfs were 3 days and 14 hours, respectively [27]. They also contrast with earlier research in Cameroon that discovered longer incubation durations for macrofilariae (5 days) [45, 46] and mfs (7 days) [26]. The difference observed might be related to an excess of carbon dioxide because earlier research indicates that variations in carbon dioxide concentrations can negatively influence the behavior of worms such as *Caenorhabditis elegans* by lowering their motility [47]. Although the motility test used is popular, there might be some limitations in discriminating low motile worms from dead worms as early reported [48]. More sensitive and robust technologies, including video-, fluorescence-, metabolic enzyme-, and impedance-based instruments, might have been effective in improving survival rates [49].

Then, the macrofilaricidal and microfilaricidal activities of the different aqueous extracts of the plant were evaluated. First, single-concentration screenings of 1 mg/mL and 0.5 mg/mL were carried out on Mfs and mfs, respectively. The bark aqueous extract was more effective than the leaf and root extracts on both stages of the parasite. This may be due to the presence of more active ingredients in this bark extract than other water extracts. Our findings vary from those of a previous study that found that extracts of *Khaya senegalensis* leaves and bark had more intriguing filaricidal activities than root extracts [29]. The extraction method and the solvents used as well as the chemical composition of the plant might explain the discrepancy observed.

A concentration-dependent investigation of the filaricidal activity of the bark extract revealed interesting macrofilaricidal and microfilaricidal efficacies but lower compared to flubendazole and ivermectin, respectively. This could be related to an interaction between the bioactive compounds that decreases the filaricidal properties of the plant extract. These findings are consistent with a previous study that found flubendazole to be more active on *O. ochengi* than hydromethanolic extracts of *Khaya senegalensis* leaves and bark after 48 h incubation [27]. However, our findings contrast with those of prior research in which the methanolic extract of *Indigofera tinctoria* was shown to be more active than ivermectin on the adult male of *O. ochengi* after 72 hours of incubation at 37°C [50]. The disparity observed might be attributed not only to the kind of extract used, its phytochemical content, and the time of incubation but also to the reference medication utilized, which was not appropriate for this model because ivermectin is known to be less active in adult worms than mfs [28, 51]. Indeed, ivermectin binds to chloride-glutamate-gated channels in muscle and neuronal cells of the mfs with a high affinity. Its attachment to these channels causes the parasite's membrane to hyperpolarize, resulting in paralysis. However, in female worms, it mostly exerts an embryostatic action [28].

In the pharmaceutical field, the therapeutic efficacy of a potential drug should be balanced with the toxicity to determine its selectivity profile and thus its chances of being developed as a safe drug. Interestingly, the LD_50_ of the bark aqueous extract of *C. nigricans* was found higher than 2000 mg/kg on the animal model used with no signs of adverse reactions and mortality during the 14-day observation period. This extract thus falls under category 5 of nontoxic chemicals, according to the OECD with a great selectivity index. These findings are comparable to those of previous studies, which indicated an LD_50_ of >2000 mg/kg after rats were given water extracts of *Combretum micranthum* leaves [52]. Flubendazole also has a low oral toxicity with an LD_50_ greater than 5000 mg/kg in rats as previously reported [53].

The phytochemical study highlighted various compounds in leaf, root, and bark extracts. However, saponins, deoxyoses, and polyphenols including tannins, phenolic acids, anthocyanins, and flavonoids were particularly detected in the bark extract, but no alkaloids were found. These results contrast with another study that found alkaloids, terpenoids, and tannins in the aqueous extracts of the aerial portions of *Combretum nigricans* [54]. The quantitative investigation of these metabolites in the barks showed a significant increase in flavonoids, condensed tannins, and phenolic acids compared to the leaf aqueous extract (Table 5). Previous work found that certain phenolic acids such as ellagic and gallic acids possess anthelmintic properties on *O. ochengi* and *C. elegans* worms [55]. These findings are consistent with an earlier work that found significant levels of phenolic substances, condensed tannins, and flavonoids in aqueous extracts of *P. guajava* stem barks [56].

To elucidate the biochemical changes associated with the filaricidal activity of the bark extract, different markers of oxidative stress were evaluated in the homogenates of adult male worms of *O. ochengi* in comparison with the flubendazole action. To the best of our knowledge, very little information is available on the mechanisms of anthelmintic inhibition of medicinal plants, particularly on the parasite *O. ochengi*.

NO is a prooxidant compound produced by oxidation of the N-terminals of L-arginine under the action of inducible nitrite oxide synthase (iNOS) in cells. In helminths, it plays an important role in neurotransmission, muscle relaxation, and sexual maturation of male parasites [57]. At all nematocidal concentrations, we observed an increase in NO levels with flubendazole and the aqueous bark extract. This might be due to the fact that flubendazole and the bark extract activate the iNOS gene, causing an increase in NO generation in the worm cells. This is consistent with prior findings which showed that a NO-rich environment affects mitochondrial activity in helminths such as *Schistosoma japonicum* [58]. Indeed, NO may combine with the superoxide anion to generate peroxynitrite (HOONO), a potent, widely dispersed oxidant capable of causing damage to a wide range of organic molecules, most notably mitochondrial proteins [59].

A decrease in CAT activity with the bark extract and flubendazole was also noted. The inhibition of worm motility linked to the decrease in CAT activity in our study could be explained by excessive production of H_2_O_2_ due to a high level of superoxide dismutase (SOD) activity in the organism. CAT is an enzyme that converts the H_2_O_2_ into oxygen and water necessary for detoxification. Its activity complements that of SOD which transforms the superoxide anion into H_2_O_2_ [59]. Three types of CAT have been characterized in nematodes: the cytosolic (Ctl-1), peroxisomal (Ctl-2), and tissue-specific (Ctl-3) isoforms [60]. Our results are similar to previous studies which suggest that low levels of CAT in *Onchocerca* strongly promote parasite susceptibility and death [61].

Our findings also revealed a nonsignificant rise in MDA content across all treated groups. We may consequently conclude that the bark extract and flubendazole both promote lipid peroxidation, resulting in cell lysis. In eukaryotic organisms in general, biological membranes are rich in polyunsaturated fatty acids which are very sensitive to oxidation and may release products such as MDA that can react with proteins and DNA. Previous studies reported that prooxidant molecules such as zinc oxide nanoparticles at high concentrations stimulate MDA production in helminths [62]. Such molecules might be induced by the bark extract in the worms.

Aside from the rise in MDA, there was also an increase in GSH content. Previous research has found that decreased GSH levels affect the resilience of certain nematodes [63]. The increase in GSH could therefore be seen as an adaptive response put in place by the parasite to curb oxidative stress but which is not sufficient to compensate for stress already present.

## 5. Conclusion

From this study, it appears that the aqueous extracts of *Combretum nigricans* possess macro- and microfilaricidal properties on *Onchocerca ochengi* after short incubation periods and more significant activities were recorded with the bark extract although lower compared to flubendazole and ivermectin. The bark aqueous extract and flubendazole exert their antioxidant activities by increasing the NO, MDA, and GSH contents and reducing the activity of CAT. In addition, phytochemical analysis showed high contents of phenolic acids, condensed tannins, flavonoids, and saponins. All studied extracts were not hazardous to albino rats when a single dosage of 2000 mg/kg was administered for 14 days. The findings support the use of *Combretum* species in the treatment of onchocerciasis. However, further research might examine the impact of this plant on ivermectin-resistant strains, free nematodes like *Caenorhabditis elegans*, and other biochemical markers including carbohydrate metabolites and antigenic proteins in helminths to have a better knowledge of its biological activity. It may be also premature to reject any harmful effect on the organism. More toxicological research is therefore required.

## Figures and Tables

**Table 1:** Yields of crude aqueous extracts of *Combretum nigricans*.

Plant parts	Extraction type	The dry mass of the plant sample in g	Filtrate volume in mL	Mass of crude extract in g	Yield in %
Leaves	Decoction	20	55	1.3	6.5
Barks	Decoction	20	100	2	10
Roots	Decoction	20	50	1	5

**Table 2:** Catalase activities (CAT) and the contents of reduced glutathione (GSH), nitric oxide (NO), and malondialdehyde (MDA) in the homogenates of adult male worms treated with the aqueous extract of the barks of C. *nigricans* and flubendazole.

Groups	Concentration (*μ*g/mL)	NO (mM/mL)	CAT (*μ*M/Min)	GSH (*μ*mol/mL)	MDA (*μ*M/mL)
Normal	—	17.7 ± 13	100 ± 36	1.54 ± 0.005	0.90 ± 0.00
Flubendazole	300	164 ± 21.8^∗∗∗^	32 ± 16.5^∗^	2.60 ± 0.527	2.31 ± 0.00
Bark aqueous extract	300	33.7 ± 38.4	135 ± 7.76	1.72 ± 0.130	1.54 ± 0.00
	700	172 ± 13.5^∗∗∗^	88 ± 32	3.31 ± 0.891	5.01 ± 0.00

The obtained data were presented as mean values ± SEM. Comparisons between groups were performed using one-way analysis of variance (ANOVA) followed by Dunnett's multiple comparison test. ^∗^*p* < 0.05, ^∗∗∗^*p* < 0.001 when compared to the normal or untreated group.

**Table 3:** Effects of aqueous extracts of leaves, trunk bark, and roots of *Combretum nigricans* on some physiological parameters in rats.

Physiological parameters	Untreated group	Treated group (2000 mg/kg)
Piloerection	+	+
Noise sensitivity	-	-
Appearance of stools	-	-
Drowsiness	+	+
Motility	-	-
Weight loss	-	-
Mortality	0	0

+: present; -: absent.

**Table 4:** Qualitative phytochemical screening of aqueous extracts of *Combretum nigricans*.

Secondary metabolites	Leaves	Roots	Barks
Alkaloids	-	+	-
Tannins	+	-	+
Saponins	+	-	+
Phenolic acids	+	+	+
Flavonoids	-	-	+
Triterpenes	+	-	-
Glucosides	+	-	-
Anthocyanins	-	-	+
Deoxyoses	+	-	+

+: present; -: absent.

**Table 5:** Contents of phenolic acids, condensed tannins, flavonoids, and saponins in the different aqueous extracts of *Combretum nigricans*.

Aqueous extracts	Phenolic acids (mg GAE/g DM)	Condensed tannins (mg CE/g DM)	Flavonoids (mg RE/g DM)	Saponins (mg)
Barks	98.7 ± 0.402^∗∗∗^	545 ± 0.333^∗∗∗^	361 ± 1.35^∗∗∗^	0.0612 ± 0.0139
Leaves	56.5 ± 0.261	475 ± 2.52	226 ± 0.206	0.0506 ± 0.0241
Roots	8.79 ± 0.292^∗∗∗^	588 ± 4.63^∗∗∗^	345 ± 1.49^∗∗∗^	0.00 ± 0.00

The obtained data were presented as mean values ± standard error of the mean (SEM). Comparisons between the means were carried out using an ANOVA test followed by Dunnett's multiple comparison test against the leaf extract. ^∗∗∗^*p* < 0.001. GAE: gallic acid equivalent; CE: catechin equivalent; RE: rutin equivalent; DM: dry matter.

## Data Availability

The data used to support the findings of this study are available from the corresponding author upon request.

## Abbreviations

CAT: Catalase
CE: Catechin equivalent
DM: Dry matter
GAE: Gallic acid equivalent
GSH: Glutathione
MDA: Malondialdehyde
Mfs: Macrofilariae
mfs: Microfilariae
NO: Nitric oxide
PBS: Phosphate buffer saline
RE: Rutin equivalent
RPMI-1640: Roswell Park Memorial Institute 1640
SEM: Standard error to the mean
TBA: Thiobarbituric acid
TCA: Trichloroacetic acid
WHO: World Health Organization.
